# Patterns of admission in forensic units during the COVID-19 pandemic: a process analysis

**DOI:** 10.3389/fpsyt.2023.1339545

**Published:** 2024-01-11

**Authors:** Isabella D’Orta, François R. Herrmann, Panteleimon Giannakopoulos

**Affiliations:** ^1^Division of Institutional Measures, Medical Direction, Geneva University Hospitals, Geneva, Switzerland; ^2^Institute of Global Health, University of Geneva, Geneva, Switzerland; ^3^Department of Rehabilitation and Geriatrics, Geneva University Hospitals and University of Geneva, Geneva, Switzerland; ^4^Department of Psychiatry, University of Geneva, Geneva, Switzerland

**Keywords:** acute psychiatric care, COVID-19 pandemic, forensic, process analysis, personality disorder

## Abstract

**Introduction:**

The impact of COVID-19 pandemic on forensic service practice remains matter of debate. Increased rates of anxiety, depression, and exacerbation of psychotic symptoms were reported in the early phases of the pandemic among detained persons. However, longitudinal analyses in medium-security hospitals taking into account the whole pandemic period led to mitigated results.

**Methods:**

This report examines the evolution of the type (voluntary versus involuntary) and reason of admissions, length of stay, history of outpatient care, short-term seclusion hours for aggressive behaviors, and clinical diagnoses for detainees needing acute psychiatric care during the COVID-19 pandemic in the sole secure ward located in the central prison of Geneva, Switzerland. To determine the general trend of the processes over time we applied a combination of process analysis with run chart plotting, and fractional polynomial regression.

**Results:**

Run tests showed that the proportion of cases with personality disorders, substance use disorders (SUD), and previous outpatient care tended to increase during the COVID pandemic with subsequent decrease to reach the pre-COVID values. This was also the case for depressive symptoms as reason for admission. The proportion of involuntary admission showed a steady increase both during the COVID and post-COVID time periods. In contrast, short-term seclusion hours decreased during the COVID pandemic followed by a return to their pre-COVID values. Regression models revealed that the COVID pandemic was associated with a significant increase in the rates of inmates with personality disorders and SUD admitted for forensic care explaining 36 and 41% of their variance.

**Discussion:**

These data support the idea that, in terms of acute care needs, the COVID-19 pandemic was associated with increased needs for acute forensic care that concerned detainees with personality disorders characterized by increased levels of impulsiveness, decreased tolerance to frustrations, loss of control, increased extraversion and frequent SUD comorbidity.

## Introduction

The COVID-19 pandemic was declared on 11 March 2020 by the World Health Organization, producing tremendous repercussions on daily living, mostly for vulnerable populations. In an attempt to contain the disease transmission and the overcrowding of the health system, governments imposed social restriction measures ranging from strict lockdown measures to stay- at-home orders and even nighttime curfews, and recommended behaviors (physical distancing). The intensity of social restrictions fluctuated as a function of the number of new infections. The repercussion of the COVID-19 related measures on mental health has been exhaustively studied in the general population compared to psychiatric patients with conflicting and, in some cases, surprising data (1–7). In general population, prevalence of depression and anxiety increased during the initial phase of the pandemic (8, 9). However, longitudinal studies reported no or very modest changes in these symptoms compared to pre-pandemic levels (10–13). A review on 25 studies assessing this issue in general population concluded that the psychological impact of the COVID-19 lockdowns was small on average (14). Some studies indicated that psychological response to the pandemic should be more intense in vulnerable individuals with severe mental health disorders (15–17). However, other large-scale studies that included pre-pandemic data showed that while psychiatric patients experienced larger mental health burden overall, the impact of COVID-19 stressors was equal or even lower in this group compared to non-clinical samples (4–6, 18–20). A systematic review published in 2022 pointed to the heterogeneity of the results and concluded the worsening of symptoms in psychiatric samples during the first phase of COVID-19 pandemic was inconsistent and mostly concerned specific conditions such as obsessive-compulsive and eating disorders (7).

The COVID-19 pandemic had also major impact on forensic service practice [for review see (1–5)]. Detainees were exposed to live in confined spaces (overcrowded, poorly ventilated and often insanitary environments) with poorer physical heath and increased isolation due to the restricted access to outside visitors. At the initial phase of the pandemic, increasing feelings of health-related anxiety, stress, and depression were reported among detainees with pre-existing mental health difficulties (21). Later studies in this field showed that prolonged lockdowns and preventive quarantine resulted in increased rates of anxiety, depression, and exacerbation of psychotic symptoms among detained persons (22, 23). However, these observations were mitigated by two more recent studies that took into account the whole period of COVID-19 pandemic. In medium-security hospitals providing log-term rehabilitation of forensic patients with severe and enduring mental disorders, incidents of violence were significantly more frequent only during the late phases of lockdown. In the same line, one among the rare longitudinal studies in this field reported that admissions to the acute ward, self-harm, and assaults did not change significantly in detained persons during the COVID-19 period (24, 25).

In Switzerland, the application of comprehensive measures including lockdown, social restriction policies and stay-at-home orders was much more flexible than in other European countries due to the high availability of intensive care facilities. As a consequence, the public health policy has been frequently amended according to the evolution of the disease transmissions without strict lockdowns or nighttime curfews. However, the various restrictions have been extended repeatedly facing the risk of subsequent waves of the disease. As a consequence, the period between March 2020 and June 2021 was characterized by the continuous presence of social restrictions of various intensity without well-defined periods of strict lockdown.

From 16 March 2020 to 17 April 2020 (first epidemiological wave) Swiss authorities decided of the most impacting policies, notably, the invitation to stay at home and the suspension of school teaching. Indeed, population was invited to stay at home as much as possible, reduce physical contact and work remotely. Classes in schools were temporarily suspended, but schools remained open and children whose parents were working in-person (especially in the healthcare domain) were allowed to attend the courses. Shops were closed and only grocery market remained open during this phase. Later on, from April 2020 to June 2021, population was still invited to stay at home, to wear masks in closed places, to reduce physical contact and to work remotely if possible, but shops were open and activities in schools and other social activities were resumed normally. The policies applied in prisons were the direct reflect of those implemented in the community. In particular, physical distancing measures and suspension of most activities implying leaving the cells were diminished. Following WHO and the Council of Europe recommendations detainees were still allowed to attend 1 h of free time outside, in the prison yard. However, during the first epidemiological phase (April 2020 to June 2021) a decrease in sport activities was observed, as well as the closure of most workshops and a drastic decrease of contacts with the outside world, especially with families but also with lawyers, social workers or other actors of their legal or social follow-up. UHPP did not suffer for any shortage of staff (medical doctors, nurses and other professionals) during the COVID-19 pandemic.

To date, the impact of this exceptional period on the use of acute psychiatric wards in prison in Switzerland has not been addressed. Our study was based on routinely collected outcome data in the sole secure ward located in the central prison of Geneva, Switzerland. We considered variables already considered in previous studies addressing the impact of COVID-19 pandemic on forensic care: number, type (voluntary versus involuntary) of admissions and length of stay (care pathway progression), previous history of outpatient care, reason of admission and ICD-10 clinical diagnosis (clinical variables), and short-term seclusion hours (restrictive interventions) (21–25). In comparison with the pre- and post-COVID-19 period, we postulated that persons with long-standing psychological vulnerability would be more likely admitted, the prevalence of depressive and anxious disorders would be higher and use of short-term seclusions lower during the period of COVID-19 related social restrictions.

Switzerland does not have a unified federal law governing compulsory hospitalizations for psychiatric reasons. Mental health laws and procedures may vary from one county to another. Following the Art. 426 of the Swiss Civil code, “a person can be placed in an appropriate institution when, due to psychological disorders, a mental deficiency or a serious state of abandonment, assistance or the necessary treatment cannot be provided to him or her in another manner.” In Geneva, a medical doctor of any specialty, having accomplished the post-graduate training (FMH diploma) is entitled to establish PAFA (i.e., Placements à des fins assistance, placements with the aim of assistance). PAFA are compulsory hospitalizations, which can last up to 40 days and that must take place in psychiatric units. The patient or one of their relatives can appeal against the decision of compulsory hospitalization, writing a request of reassessment to the civil judge.

## Methods

### Sample characteristics

The UHPP (Unité hospitalière de psychiatrie pénitentiaire) is a 15 beds unit specially designed for acute psychiatric care of detained persons from the French speaking counties and is part of a medium-security hospital located in prison. Admission to the UHPP was based on need for urgent psychiatric care because of the presence of acute depressive or psychotic symptoms, psychomotor agitation with self or others-threatening behaviors. The health care team is composed of 4 medical doctors, 35 nurses and one nurse-auxiliary. Five nurses are present during every day shift (2 between 9 p.m. and 7 a.m.). Prison staff is continuously present, usually 2–4 prison guards for shift. They guarantee the security during daily activities in the unit. Care programs are based on the integration of psychopharmacology and psychotherapeutic approaches. The vast majority of the patients receive psychotropic medication. They also systematically benefit from at least one clinical encounter with a nurse during the day and 4–5 clinical encounters with the medical doctors during the week. Group therapy, art-therapy and ergotherapy (a physical therapy aiming to reduce pain, discomfort and functional disability) are performed on a regular basis. Clinical activities take place according to the prison timetable/schedule. Patients are allowed to spend time together in a common room (2 h in the morning and 3 h in the afternoon). Following the penitentiary international rules, patients are allowed to spend 1 h of time in the yard of the unit, where a tennis table is available. Five slots per day are scheduled for smoking patients, during which they have access to a limited part of the yard. In the UHPP, individual therapies and usual clinical activities were maintained even during the period of COVID-19 related restrictions in prisons. Regarding group activities, they were canceled during the first epidemiological wave only (March–April 2020).

In order to examine the impact of COVID-19 pandemics on forensic settings of acute care, we examined retrospectively the psychiatric records of all cases admitted in UHPP between January 1st 2019 and December 31st 2022 (total number of admissions = 1,031). There are no exclusion criteria in this study. Since the main objective was to examine the COVID-19 related temporal changes in our outcome variables and in order to avoid selection biases, it was crucial to consider all of the admissions during the period of reference. The following variables were registered: number (continuous) and type of admission (voluntary versus involuntary), reasons of admission (suicidal attempts or thoughts, agitation, depressive symptoms, panic attacks, others), short-term seclusion hours for aggressive behaviors (continuous), clinical diagnoses according to ICD-10 classification, length of stay (continuous), previous history of outpatient care (binary). The reason of admission is systematically noted on the request template. The term of depressive symptoms corresponds to the reason of admission noted in the request template signed by the psychiatrist (working in another facility) who refers the patient to the UHPP. All of the ICD-10 clinical diagnoses were made at the time of admission by two independent psychiatrists blind to the scope of the study. As a routine procedure, at admission, the clinical diagnosis is made by a board-certified senior staff member. In case of admissions from an external center, the diagnosis retained was that proposed by the psychiatrist of this center in charge of the detainee. Cases with multiple diagnoses were considered in each diagnostic group separately. Each patient was assigned an identification number that was derived from the name and birth date and subsequently encrypted. Psychiatric diagnoses included adjustment disorders, bipolar disorder, depressive disorders (ICD-10 codes F32-33), anxiety disorders (F40-42), personality disorders (antisocial and borderline type), psychosis (ICD-10 codes F20-F29) and intellectual disability. In our sample, there were no other ICD-10 defined personality disorders. The comorbidity of substance use disorders was treated as binary variable.

### Statistics

To describe the evolution of different admission parameters over time we applied process analysis, with run chart plotting the observed data over a time sequence and run tests. The later tests whether a set of observations occurs randomly (the null hypothesis), that is, whether they are serially independent, by counting how many runs there are above and below the median. A run is defined as one or more consecutive data points on the same side of the median line. A small number of runs indicates a positive serial correlation; a large number indicates a negative serial correlation. Significant *p* values imply that the set of observations is not randomly distributed (26). To determine the general trend of the processes over time we applied fractional polynomial regressions (FPR) of degree 3, using the “fp” Stata command. It automatically fit the best regression models among a combination of powers of time (−2 −1 −0.5 0.5 1 2 3), that is (y = a_1_/x^2^ + a_2_/x + a_3_/√x + a_4_* √x + a_5_* x + a_6_* x^2^ + a_7_* x^3^), to predict the number of observation (y). The coefficient of determination (R^2^) is the proportion of the variance in the number of events explained by the regression models. All statistics were performed with Stata release 18.0, StataCorp, College Station, TX, United States 2023.

## Results

**Figure 1:**
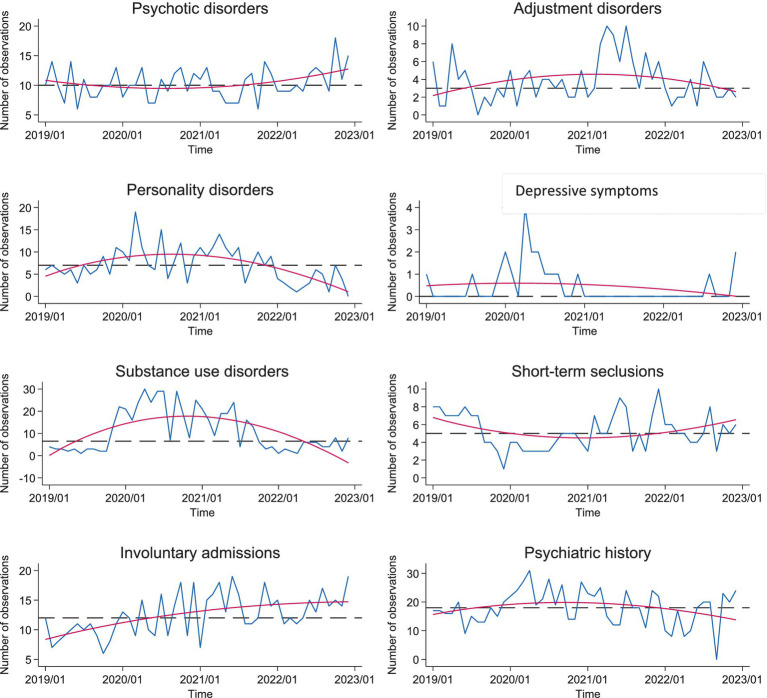
Run charts displaying (in blue) the number of observations over time (months). The horizontal dotted line corresponds to the median (in black), the red curve shows the trends according to fractional polynomial regression.

Table 1 summarizes the results of run tests and FPR analyses for all significant and two control (non-significant) variables. Among the variables tested, involuntary admissions, short-term seclusion hours, previous history of outpatient care, and depressive symptoms at admission showed a non-random distribution when comparing the time periods before, during and after the COVID pandemic. This was also the case for two ICD-10 diagnoses: personality disorders and SUD. The corresponding run charts are illustrated in Figure 1. They reveal that the proportion of cases with personality disorders, SUD and previous outpatient care tended to increase during the COVID pandemics with subsequent decrease to reach the pre-COVID values. This was also the case for depressive symptoms as reason of admission. The proportion of involuntary admissions showed a steady increase both during the COVID and post-COVID time periods. In contrast, short-term seclusion hours decreased during the COVID pandemic with subsequent return to the pre-COVID values. The FPR results revealed that only the proportion of patients involuntarily admitted, and the rate of inmates with personality disorders and SUD comorbidity significantly varied during the time frame of reference. The percentage of variance of these parameters explained by time reached 31.5, 36.07, and 43.04%, respectively (Table 1). Figure 1 showed the inverted U shape distribution of values for personality disorders and SUD as compared to the continuous increase of involuntary admissions. Occurrence of depressive symptoms, history of outpatient care and short-term seclusion hours did not reach significance in FPR models. With short term seclusion we refer to the practice where a patient is placed for a short time (few hours or maximum 24 h) in a separate and secure environment. In the UHPP this place is called intensive care room (ICR) and it is used to manage acute behavioral issues and to ensure safety of the patient. During the time spent in the ICR the patient is strictly monitored with specific protocols for vital signs, reaction to pharmacological treatments and general status.

**Table 1:** Results from the run test and the fractional polynomial regressions.

		Run test							Fractional polynomial regression	
	N	Nb of runs	Mean	Median	Nb of runs above	Nb of runs below	*Z*	*P*	*R* ^2^	*P*
Psychotic disorders	48	24	25	10	21	27	−0.1853	0.8530	12.11	0.0548
Adjustment disorders	48	22	25	3	23	25	−0.8648	0.3872	8.15	0.1477
Involuntary admissions	48	16	25	12	22	26	−2.5960	0.0094	31.50	0.0002*
Short-term seclusions	48	13	24	5	19	29	−3.3460	0.0008	11.99	0.0565
Depressive symptoms	48	13	21	0	14	34	−2.7787	0.0055	4.41	0.3623
Personality disorders	48	15	24	7	19	29	−2.7353	0.0062	36.07	<0.0001*
Substance use disorders	48	8	25	7	24	24	−4.9605	<0.0001	43.04	<0.0001*
Psychiatric history	48	14	25	18	23	25	−3.2033	0.0014	13.71	0.1649

The coefficient of determination (R^2^) is the proportion of the variance of the number of events explained by the regression models. Nb, number. See text for details. *variables showing statistically significant associations with COVID-19 pandemic.

## Discussion

The present data indicate that detainees with personality disorders and SUD were more frequently admitted in acute forensic settings during the COVID-19 pandemics compared to the pre- and post-COVID-19 years. In contrast to what one could expect, the rate of admission for adjustment and affective disorders remained stable during this time frame. Detainees with history of outpatient care were also more prone to be admitted in these settings during the COVID-19 pandemics, yet, in agreement with previous observations in non-forensic samples (4, 5, 18–20, 27), this increase was not statistically significant in regression models. Similar observations were made for the short term seclusions that tended to be less frequently used during the period of COVID-19 related social restrictions.

To our knowledge, this is the first study attempting to explore whether the COVID-19 pandemic was related to significant changes in the pattern of forensic admissions for acute psychiatric care in Switzerland. To date, only two samples in forensic settings examined the longitudinal evolution of care needs during the whole duration of COVID-19 pandemic (24, 25). As already reported by Koch et al. (24) we did not observe a significant increase of the number of admissions in our acute ward during the COVID-19 pandemic. This may be related to the fact that since prison is a more controllable place, isolation rules are easy to regulate and do not induce an overall psychological burden sufficiently severe to induce increased needs for inpatient psychiatric care. In the same line, adjustment and affective disorders, the two main pathologies that were thought to increase due to the impairments in private and everyday life and unpredictable course of the contaminations, were not more frequent in the present sample after the outbreak of the pandemic. The same is truth for self- and other-threatening behaviors including suicidal ideation and attempts. These observations parallel several longitudinal reports showing that the global psychological impact of the pandemic was modest in the general population (10, 12–14, 17). In another longitudinal study of forensic mental health services, Puzzo et al. (25) reported increased use of long-term seclusions during the three strict lockdowns decided by the government in United Kingdom. Physical and non-physical assaults to service users and incidents of self-harm increased only in the third lockdown (May 2021). Two main differences could explain the discrepancy of these results. First, both longitudinal studies (24, 27) concerned medium and low security rehabilitation settings with very long duration of stays. Second and in respect to the data from United Kingdom, strict lockdown with major restriction of social contacts was not decided in Switzerland. This policy could be associated with a decreased rate of physical and non-physical aggressions among forensic patients.

One main finding of the present study is the increased rate of admission of detainees with personality disorders and SUD during the COVID-19 pandemic. This tendency was not only detected by our run analysis but also confirmed in regression models with quite substantial percentages of variance explained by the occurrence of the COVID-19 outbreak alone. Unlike the rate of involuntary admissions that increased significantly from the end 2019 to the end of 2022, the increased use of acute forensic wards from patients with personality disorders and SUD is strictly related to the COVID pandemic period. In contrast to adjustment and affective disorders as well as psychotic episodes needing acute psychiatric care, borderline and antisocial personality disorders are long-lasting conditions that are characterized by increased levels of impulsiveness, decreased tolerance to frustrations and loss of control, but also increased extraversion with search for social contacts. One could speculate that the social restrictions and daily life constraints imposed by the pandemic had a greater psychological impact on these detainees compared to their group of reference. Several previous contributions pointed to the detrimental effect of COVID-19 related restrictions on the mental health of patients with borderline personality disorder. They displayed an increase of distress and depressive feelings, and non-suicidal self-injuries (28–32) but also increased rate of admission for inpatient care (33). Reports on antisocial personality mostly focused on the poor compliance with the COVID-19 rules of protection among these patients, in particular in case of lack of empathy and callous behavior (34–36). Other contributions stressed the role of patients with dual diagnosis, namely borderline personality and SUD with increased rate of suicidal attempts in the second way of COVID-19 contaminations, steady increase of anxious and depressive symptoms (37–40), as well as increased use of acute psychiatric wards (41, 42). In conjunction with these community-based reports, our findings reveal an increased use of acute forensic care in prison by detainees with borderline and antisocial personality disorders. As already postulated, the most plausible explanation for this finding resides to their vulnerability to social isolation and distress facing restrictive rules (36). Of importance, more than 85% of inpatients with personality disorders also displayed SUD, stressing the relative weight of dual diagnosis in the use of acute forensic wards during the COVID-19 pandemic.

Strengths of the present study include the admission of all cases in the same unit of acute psychiatric care in prison that decreases the variability in the admission criteria, use of strict statistical criteria combining run tests and FPR to detect the significant fluctuations as a function of the COVID-19 pandemic and consideration of the whole period of COVID-19 measures without artificial reference to limited lockdown periods. Several limitations should, however, be mentioned. The limited sample did not allow for studying the effect of the pandemic on cases with SUD without other psychiatric comorbidities as well as other pathologies characterized by decreased self-control such as attention deficit syndrome. Clinical diagnosis was carried out by two independent clinicians blinded to the aim of the study. Standardized diagnostic questionnaires were not used in order to be close to a real-life situation. The assessment of previous outpatient care outside the Geneva County was also made by self-report and could be biased. We examined a restricted set of data that concerned care pathway, clinical and restrictive interventions-related variables. The impact of COVID-19 pandemic on other parameters such as detention period, type of crimes, and physical comorbidities was not addressed. These observations concern a specialized unit of forensic psychiatry located in prison and not in a psychiatric hospital. The effect of COVID-19 pandemic may be radically different in low security settings that guarantee better social support and access to reliable information (24). In the same line, our findings cannot be compared with those made in long-term rehabilitation centers for patents with court-ordered treatments. A comparison of the COVID-19 related evolution of parameters such as treatment outcome, psychotropic medication use, perceived mental health, and prosocial behaviors is needed to gain a more in depth understanding of the impact of this exceptional period on the mental health of detainees.

## Data availability statement

The raw data supporting the conclusions of this article will be made available by the authors, without undue reservation.

## Ethics statement

The studies involving humans were approved by Commission Cantonale d’Ethique de la Recherche (CCER), Service de la pharmacienne cantonale, Département de la santé et des mobilités (DSM), Republique et canton de Geneve. The studies were conducted in accordance with the local legislation and institutional requirements. Written informed consent for participation was not required from the participants or the participants’ legal guardians/next of kin in accordance with the national legislation and institutional requirements.

## Author contributions

ID’O: Conceptualization, Data curation, Investigation, Writing – original draft. FH: Formal analysis, Methodology, Software, Writing – original draft. PG: Conceptualization, Methodology, Supervision, Writing – original draft.
